# Coats' Disease-Related Macular Edema Treated with Combined Aflibercept and Laser Photocoagulation

**DOI:** 10.1155/2017/2824874

**Published:** 2017-12-12

**Authors:** Wen-Shi Shieh, Gaurav K. Shah, Kevin J. Blinder

**Affiliations:** The Retina Institute, 1600 S. Brentwood Blvd., Suite 800, St. Louis, MO 63144, USA

## Abstract

**Purpose:**

To describe the clinical response of refractory macular edema associated with Coats' disease following treatment with aflibercept and laser photocoagulation.

**Methods:**

Case report.

**Results:**

A 17-year-old female presented with decreased vision of the left eye. Ophthalmic exam demonstrated intraretinal hemorrhages and exudation with associated edema centrally. Angiographic evaluation revealed central leaking microaneurysms and peripheral capillary dropout. These findings and a systemic work-up that yielded an incidental Factor V Leiden mutation lead to a diagnosis of Coats' disease. Initial treatment consisted of laser photocoagulation and intravitreal bevacizumab but with poor response. Switching to intravitreal aflibercept resulted in resolution of the refractory macular edema and improvement of visual acuity to 20/25 in the left eye.

**Conclusion:**

We describe a case of refractory macular edema which responded more favorably to intravitreal aflibercept compared with bevacizumab when combined with laser photocoagulation in a patient with Coats' disease.

## 1. Introduction

Coats' disease (also known as retinal telangiectasis) is characterized by abnormal telangiectasias and aneurysms of retinal vessels leading to retinal exudation and potential serous retinal detachment [1]. This condition generally has a unilateral presentation and affects males more than females with no ethnic or geographic associations [2]. Adult-onset variants of Coats' disease have also been reported with a spectrum ranging from milder forms limited to macular abnormalities to findings identical to that in children [3]. We present a case report of a female patient with clinical findings consistent with Coats' disease and its clinical course including treatment of refractory macular edema.

## 2. Case

**Figure 1:**
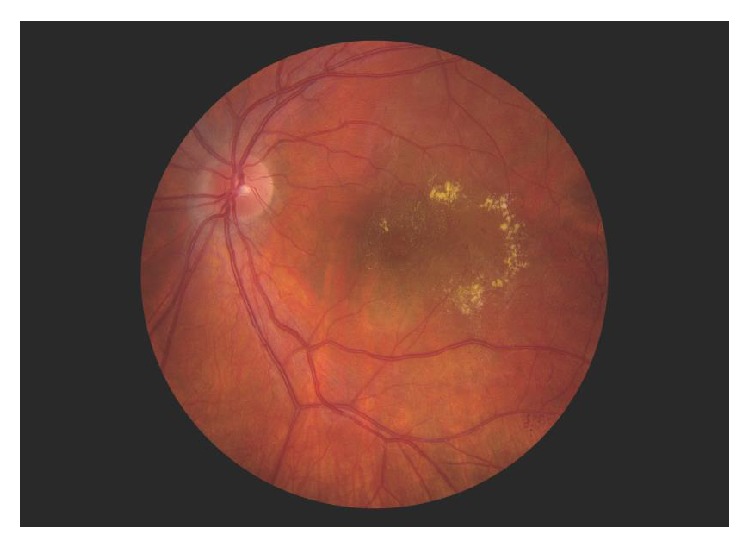
Fundus photograph of the left eye showing central exudation and intraretinal hemorrhages in the temporal macula.

**Figure 2:**
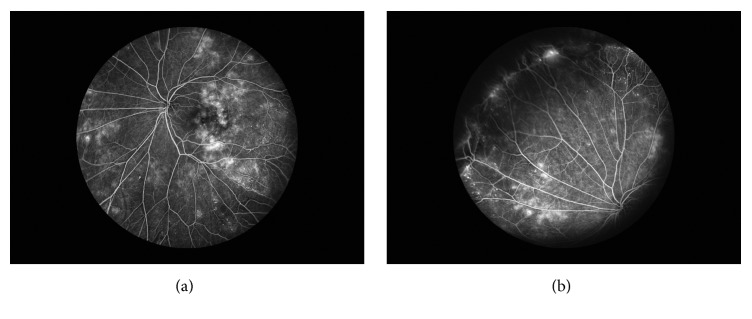
Wide-field fluorescein angiography of the left eye highlighting numerous microaneurysms and associated leakage within the macula (a) and peripheral capillary dropout (b).

**Figure 3:**
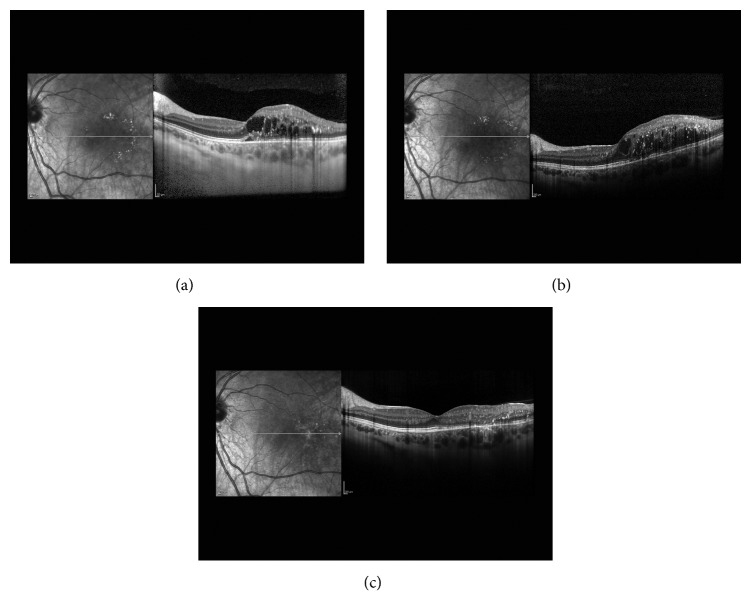
(a) Initial OCT of the macular with intraretinal edema and subretinal fluid. (b) Persistent macular edema despite initial peripheral laser photocoagulation and three intravitreal bevacizumab injections at 3 months. (c) Resolution of central macular edema at 12 months after repeated intravitreal aflibercept injections.

A healthy 17-year-old female presented with blurred vision in her left eye. Visual acuity was 20/20 and 20/40 in the right and left eyes, respectively. Fundus examination of the left eye showed intraretinal hemorrhages and exudation with associated edema centrally (Figure 1). Wide-field fluorescein angiography of the left eye revealed central microaneurysms with leakage and capillary nonperfusion peripherally (Figure 2). Spectral domain optical coherence tomography (OCT) initially demonstrated macular edema and subretinal fluid (Figure 3(a)). A work-up yielded normal CBC, PT, aPTT, and INR. Although coagulopathy studies were negative for lupus anticoagulant, anti-cardiolipin antibody, and normal levels of homocysteine, Protein S, and Protein C, a Factor V Leiden mutation was incidentally found.

The patient was diagnosed with Coats' disease and treated initially with peripheral laser photocoagulation and intravitreal bevacizumab. Despite a series of three monthly intravitreal bevacizumab injections, macular edema persisted at 3 months (Figure 3(b)). A greater response was noted upon switching to intravitreal aflibercept and the patient received 5 total injections at an interval of every 4 to 6 weeks. Additional focal laser to noncenter involving macular edema was also applied at month 10; however, the improvement of macular edema on OCT was most pronounced following two additional monthly injections of intravitreal aflibercept (Figure 3(c)). At 12-month follow-up, visual acuity improved to 20/25 without any further recurrence of edema.

## 3. Discussion

Early stages of Coats' disease involve telangiectasis in the temporal macula and mid-periphery. Retinal edema and accumulation of lipid exudates in the macula are common causes for vision loss. In later stages, proliferation of retinal pigment epithelial cells in the subretinal space can lead to fibrosis and retinal detachment. The treatment of choice has been laser photocoagulation and/or cryotherapy in early stages. In advanced stages, retinal detachment repair or enucleation for a blind, painful eye may be necessary [4]. For our patient, the differential diagnoses included a nonischemic central retinal vein occlusion or macular telangiectasia. However, angiographic evidence of peripheral avascular retina, microaneurysms largely localized in the temporal macula, and associated clinical findings of exudation with macular edema are most consistent with Coats' disease.

Anti-vascular endothelial growth factor (VEGF), namely, intravitreal bevacizumab, has been reported as an effective therapy either alone or combined with other treatment modalities for subretinal fluid and exudation in Coats' disease [5–8]. For our patient, focal laser and switching anti-VEGF therapy to intravitreal aflibercept helped to reduce the refractory macular edema. To the best of our knowledge, only one other case report exists of Coats' disease-associated macular edema responding more favorably to aflibercept injections after initial treatment with ranibizumab and argon laser [9].

In summary, studies have shown elevated levels of VEGF in patients with Coats' disease as well as correlations between VEGF concentrations and the severity of disease [10, 11]. Although several studies highlight the efficacy of adjunctive bevacizumab injections combined with laser vascular ablation, we present a case of Coats' disease where treatment with aflibercept was effective in a patient with refractory macular edema unresponsive to bevacizumab and laser photocoagulation.
